# Feasibility and Acceptability of a Culturally Tailored Website to Increase Fruit and Vegetable Intake and Physical Activity Levels in African American Mother-Child Dyads: Observational Study

**DOI:** 10.2196/12501

**Published:** 2019-03-22

**Authors:** Alicia Chung, Barbara Wallace, Monica Stanton-Koko, Azizi Seixas, Girardin Jean-Louis

**Affiliations:** 1 New York University School of Medicine New York, NY United States; 2 Teachers College, Columbia University New York, NY United States; 3 Mercy College Dobbs Ferry, NY United States

**Keywords:** childhood obesity, eHealth, social justice

## Abstract

**Background:**

African American youth (aged 8-14 years) do not adhere to national dietary and physical activity guidelines. Nonadherence to these recommendations contributes to disproportionate rates of obesity compared with their white counterparts. Culturally tailored electronic health (eHealth) solutions are needed to communicate nutrition and physical activity messages that resonate with this target population.

**Objective:**

This study aimed to identify the impact of exposure to a website hosting culturally tailored cartoons to inspire fruit and vegetable uptake and physical activity levels in African American mother-child dyads.

**Methods:**

Statistical analysis included paired sample *t* tests to evaluate knowledge gains, self-efficacy, and readiness to change. Adapted items from Prochaska’s Stages of Change toward the following 4 behaviors were assessed with pre- and posttest surveys: (1) fruit and vegetable selection on my plate, (2) meal preparation, (3) fruit and vegetable selection outside of home, and (4) physical activity. Open-ended comments on videos from mother-child dyads were used to determine user acceptance. Observations of repeated responses during content analysis informed coding and development of key themes.

**Results:**

A final sample size of 93 mother-child dyads completed the study. Mothers reported significant improvement from precontemplation or contemplation stages to preparation or action stages for (1) fruit and vegetable selection on her plate (*P*=.03), (2) meal preparation for her family (*P*=.01), (3) fruit and vegetable selection outside the home (*P*<.001), and (4) physical activity (*P*<.001). Significant improvements were found in knowledge, stage of change, and self-efficacy for the 4 target behaviors of interest (*P*<.001). Children’s open-ended commentary reported vicarious learning and positive character identification with brown-skinned cartoons exhibiting healthful food and exercise behaviors. Mothers commented on the lack of accessible produce in their neighborhoods not depicted in the cartoon videos.

**Conclusions:**

Culturally adapted cartoons that incorporate tailored preferences by African American families, such as race or demography, may help increase adherence to target health behaviors when developing eHealth behavior solutions.

## Introduction

### Background

Compared with their white counterparts (14%), African American (22%) children and adolescents are disproportionately affected by childhood obesity [1]. Poor adherence to the US Dietary Guidelines and physical activity recommendations has been consistently found to be major risk factors for obesity among minority youth, particularly among adolescents aged 12 to 19 years [2-5]. In a nationally representative sample of children and adolescents, black adolescents were the least likely to adhere to daily dietary targets of 5 or more servings of fruits and vegetables and more than 1 hour of physical activity. Nearly none (3%) of the children adhered to dietary fruit and vegetable targets, whereas 71% met physical activity goals [5]. Given the low adherence to dietary guidelines compared with physical activity, this study focused on 3 of the 4 target behaviors around healthy eating behaviors in African American youth.

Nonadherence to dietary recommendations can be attributed to a lack of tailored interventions that address the contextual, structural, and family environments that influence food choices. Inclusion of the family, especially the mother who generally makes food purchasing decisions for the family, serve as a crucial area to intervene to address food choice (proximally) and obesity (distally). Customized framing of dietary messages for mother and child aims to tailor messages that match the receivers’ race, culture, ethnicity, demography, socioeconomics, or other preferences [6]. Tailored messages aim to ensure they are well received and accepted by the end user.

Programs that address multiple levels of the socioecological model have led to the successful engagement of minority communities [7]. This framework can be accounted for the personalization of tailored messaging to black mothers and their children. The levels of the socioecological model include intrapersonal (knowledge, attitudes, and beliefs), interpersonal (family and friends), environmental (physical structures in urban design), community (neighborhood access and safety), and policy levels (federal programs that affect food price, access, and quality). At the environmental level, structural factors such as limited access to healthy foods in one’s neighborhood (ie, food desserts for healthy foods or food swamps of fast-food chains) presents the need for novel yet sustainable solutions to increase healthier food choice (proximal outcomes) and in turn reduce the high prevalence of obesity among African American adolescents (distal outcomes) [8]. At the interpersonal level, family influence plays an instrumental role in food choices and eating behaviors. Intrapersonal factors are influenced by education as well as messaging in one’s surrounding environment. Thus, family-based interventions targeting both mother and child are needed to influence multiple levels of the family structure to reinforce messaging of key health behaviors.

Healthy People 2020 health communication and health information technology objectives aim to increase the proportion of social marketing in health promotion and health literacy communications available through media technology [9]. Nearly 75% of African American adults own a mobile phone device [10], serving as an opportune channel for disseminating health messages. Culturally tailored dietary education provided via digital media to mothers can effect wholesale change in a family’s eating habits and food choice [11]. Tailored digital solutions may lead to the adoption of obesity prevention behaviors in African American children and parents [12,13].

### Objective

We argue that to optimize uptake and adherence to culturally tailored electronic health (eHealth) nutrition and physical activity messaging interventions among black youth, it is critical to embed family-based food preferences and their contextual landscape that may spur self-referential thinking that resonates with the viewer. Incorporating these preferences is crucial to ensure receptiveness and behavior change [14]. Prior websites and gaming interventions have increased 100% fruit juice intake, vegetable consumption, and physical activity patterns and reduced body weight in African American youth [15-17]. Behavioral interventions targeting the African American mother and child as a family unit have not yet been explored.

This study aimed to assess the feasibility and acceptability of a culturally tailored website hosting cartoons to increase willingness to increase fruit and vegetable selection and physical activity among African American mother-child dyads.

## Methods

### Procedures

This study employed an observational study design. The Web-based trial began with the website storyboard, an outline of the digital story elements in a website [18]. Cartoons were created using the GoAnimate platform. Character dialogue in the scripts, developed and edited by health educators and mothers, ensure preferences of website content. Storytelling narrative via cartoon storyboards may be effective for influencing diet and physical activity change in minority youth [15-17]. Culturally appropriate cartoons, where health behaviors are modeled in a virtual representation of the viewer’s self, employ role modeling constructs from Bandura’s Social Cognitive Theory, which has led to successful behavior change in at-risk populations [19]. This theory served as the theoretical underpinning of this study and evidence-based approach for adherence of the target behaviors.

Final videos were uploaded from GoAnimate to YouTube and then onto the intervention website. The study setting was a website portal that included an embedded link to the study survey. Subjects were able to move freely from the study survey to the new website portal (for exploring the website and watching the 9 videos) and back to the survey to answer final questions.

### Website

The website portal included an open-access website with dietary information and pictures following the US Department of Agriculture’s MyPlate guideline introduced under the former First Lady, Michelle Obama. Moreover, 2-min cartoon videos on the following 4 target behaviors were included: (1) food choices for their plate, (2) meal preparation, (3) food choices when outside of the home, and (4) physical activity. Cartoons were depicted in the grocery store, school, home, and playground (see Multimedia Appendix 1 for a screenshot of one of the cartoons). Storylines were told from the mother’s, child’s, and teacher’s perspective. Scripts were developed by health educators and finalized by the principal investigator.

### Participants

Eligibility criteria included being female, aged 25 years or older, having at least a high school education, being the mother of a child between the ages of 8 to 14 years, and identified as African American. One child per mother was permitted. Participants were recruited via Web-based social media such as Facebook, electronic mail distribution lists, and Web-based Mommy blogger groups. The sampling frame included African American mothers with children in the United States. Flyers were posted at faith-based organizations and community centers throughout the New York City area. Participants were compensated with an opportunity to win a US $300 prize, US $200 prize, or US $100 Amazon gift card lottery prize. All study activities were approved by the Teachers College Institutional Review Board in 2012. Data collection was conducted from January to April 2013.

### Measures

Research measures and instruments included an open electronic survey questionnaire via Qualtrics. The internal consistency of survey items for mothers’ global stage of change, knowledge, self-efficacy, and evaluation of website for the 4 behaviors before and after watching the 9 2-min cartoon-like videos was .951 (Cronbach alpha), and .898 (Cronbach alpha), respectively. Items were not randomized or alternated. Study participants created an alphanumeric unique identifier when beginning the pre- and posttest surveys. The survey was displayed on 10 screen pages, with 10 items displayed per page. Adaptive questioning was used with conditional response options for certain demographic and personal health background questions to reduce the number of questions asked, preserving only relevant question items. Respondents were able to review responses with a back button and review survey completion progress with a status bar. Nonresponse options were included, and all survey quantitative items were set to force-response settings. Qualitative feedback was obtained from open field response options.

Mothers viewed the website and watched the cartoons with their child. Quantitative questions asked self-assessed knowledge, self-efficacy, and stage of change in executing the target behaviors. Mothers reported their child’s rating of the videos for motivating their personal behavior change with open-ended question responses. The purposive sample completed the survey on a voluntary and confidential basis (ie, indicating having read the informed consent for parents, including an electronic assent form for minors, as well as electronic participants’ rights) as anonymous participants. The study measure required approximately 45 to 55 min for successful completion, including 15 to 20 min to complete pre- and posttest surveys. Data storage location, time length, and name of the primary investigator were clearly visible on the study eligibility, informed consent, and study website.

### Statistical Analysis

Descriptive statistics of mothers’ income, age, education, marital status, child age, gender, school enrollment, and self-reported health status for herself and her child was reported. A mixed-methods analysis was conducted. Statistical analysis included paired sample *t* tests, independent *t* tests, and backward stepwise regression to evaluate knowledge gains and self-efficacy. Pretest and posttest questions, adapted from Prochaska’s Stages of Change Theory, aimed to determine how likely participants may be ready to perform and adhere to the following 4 target behaviors after viewing the website: (1) fruit and vegetable selection on my plate, (2) meal preparation, (3) fruit and vegetable selection outside of home, and (4) physical activity. Backward stepwise regression analysis was conducted to determine the best predictor of a high score for the website. Incomplete data were dropped from the final dataset.

### Open-Ended Feedback on Mothers’ and Childs’ Preferences for Video Improvements

Open-ended feedback captured comments on video quality and recommendations for improvement. Participants were asked to rate website and video quality based on aesthetics, literacy level, content, and recommendations for improvement. Mothers recorded their child’s feedback on the videos and preferences for improvement. Finally, observations of repeated responses during content analysis informed coding and development of key themes. Thematic categories were developed based on commonalities in responses by mother and child, corresponding to their respective questions. Debriefing questions, categories, emergent themes, and direct quotes were used to develop tables in the Results section.

## Results

### Participant Description

A total of 641 African American mothers older than 25 years with children between the ages of 8 and 14 years began the Web-based study. The final sample included 93 mother-child dyads, indicating a 14.5% (93/641) response rate for those dyads that met the study criteria and successfully completed the study questionnaires (N=93). In this study, successful completion was defined by at least 100% completion of the survey responses. Internet protocol addresses were checked to identify potential duplicate entries. Of the 93 mother and child dyads that completed the study, 65% (60/93) were single mothers and reported an annual income between US $20,000 and US $60,000. Mothers were on average aged 38 years. Of the 93 children, 54% (50/93) were female and aged 10 years on average. A total of 69% (64/93) of children attended public school. After watching the study videos, a significant portion of the sample moved from their baseline readiness to change status to preparation, action, or maintenance stage for the target health behaviors. Qualitative questions assessed website acceptance and user preferences for improvement.

### Statistical Analysis

A total of 15 paired sample *t* tests indicated significant improvement across 13 areas at the Bonferroni adjustment significance (0.05/15; *P*<.001; see Table 1). In the backward stepwise regressions, predictor variables that reported nonsignificant outcomes were removed from the model in a stepwise manner until a model with all significant predictors at *P*<.05 was obtained. Mothers who reported their children to be both overweight (beta=.109; *P*<.001) and underweight (beta=.881; *P*=.02) were predictors for higher overall ratings of the website. In addition, mothers with a lower educational level (beta=−.13; *P*=.05) or currently enrolled in school (beta=.589; *P*<.001), serving as a proxy for family socioeconomic status level, were at the significance level for being a predictor for a high global rating of the cartoons.

The results of Table 1 indicate significant improvements in mothers’ level of knowledge, self-efficacy, and global stage of change scores for each of the 4 target behaviors (*P*<.001) reported. Average stage of change results was scored on a 1 to 5 scale, corresponding to precontemplation, contemplation, preparation, action, and maintenance. Mothers reported significant improvement from precontemplation or contemplation to preparation or action for eating outside of the home and physical activity.

### Children’s Feedback and Preferences for Video Improvements

Mothers’ recordings of children’s feedback were quantified based on thematic categories to children’s open-ended responses. Participants were allowed to skip questions. Of the 93 children, 64% (60/93) positively viewed the cartoons and would recommend them. A total of 33% (30/93) children reported they learned about eating fruits and vegetables at home and away from home. In addition, 15% (14/93) found the videos on exercising educational, 9% (8/93) learned about cooking at home with mom, and 7% (7/93) wanted more examples on food choice selection. Overall, 10% (9/93) found the content boring, and 35% (33 of 93) thought the animation could be improved. Direct quotes from participants were categorized based on emergent themes (see Table 2).

Moreover, 64 out of 80 (80%) children reported that they would recommend the videos to other mothers and their children. Completion of open-ended questions was not required, and the respective n values are reflected as such. Children’s reasons for not recommending the videos are listed in Table 3.

**Table 1:** Paired sample *t* tests stage of change, knowledge, and self-efficacy for mothers’ pre- and postevaluation of the electronic health cartoon videos (N=93). Bonferroni adjustment significance (0.05/15; *P*=.003). All *P* values above .003 are considered nonsignificant and only those below .003 are considered statistically significant.

Paired sample *t* tests stage of change, knowledge, and self-efficacy for mothers		Stages of change, mean (SD)	*t* test	
			*t* value (*df*)	*P* value
**Pair 1^a^**				
	Prevideos	3.59 (1.25)	−2.15 (92)	.03
	Postvideos	3.74 (1.97)	−2.15 (92)	.03
**Pair 2^b^**				
	Prevideos	4.22 (0.82)	−7.99 (92)	<.001
	Postvideos	5.08 (1.22)	−7.99 (92)	<.001
**Pair 3^c^**				
	Prevideos	4.39 (1.22)	−10.17 (92)	<.001
	Postvideos	5.38 (.77)	−10.17 (92)	<.001
**Pair 4^d^**				
	Prevideos	3.65 (1.28)	−2.643 (92)	.01
	Postvideos	3.85 (1.10)	−2.643 (92)	.01
**Pair 5^e^**				
	Prevideos	4.37 (.11)	−8.16 (92)	<.001
	Postvideos	5.18 (.79)	−8.16 (92)	<.001
**Pair6^f^**				
	Prevideos	4.43 (1.25)	−9.31 (92)	<.001
	Postvideos	5.35 (.78)	−9.31 (92)	<.001
**Pair 7^g^**				
	Prevideos	3.00 (1.47)	−5.82 (92)	<.001
	Postvideos	3.48 (1.07)	−5.82 (92)	<.001
**Pair 8^h^**				
	Prevideos	3.94 (1.40)	−9.36 (92)	<.001
	Postvideos	5.06 (.79)	−9.36 (92)	<.001
**Pair 9^i^**				
	Prevideos	4.13 (.80)	−9.73 (92)	<.001
	Postvideos	5.20 (1.30)	−9.73 (92)	<.001
**Pair 10^j^**				
	Prevideos	3.34 (1.30)	−5.69 (92)	<.001
	Postvideos	3.70 (1.03)	−5.69 (92)	<.001
**Pair 11^k^**				
	Prevideos	4.52 (1.28)	−6.66 (92)	<.001
	Postvideos	5.23 (.84)	−6.66 (92)	<.001
**Pair 12^l^**				
	Prevideos	4.38 (1.29)	−8.07 (92)	<.001
	Postvideos	5.24 (.79)	−8.07 (92)	<.001
**Pair 13^m^**				
	Prevideos	3.40 (1.10)	−6.02 (92)	<.001
	Postvideos	3.69 (.93)	−6.02 (92)	<.001
**Pair 14^n^**				
	Prevideos	4.23 (1.01)	−9.56 (92)	<.001
	Postvideos	5.32 (.65)	−9.56 (92)	<.001
**Pair 15^o^**				
	Prevideos	4.33 (1.13)	−10.556 (92)	<.001
	Postvideos	5.30 (.66)	−10.556 (92)	<.001

^a^Pair 1: Mother’s stage of change for fruits and vegetables selection on her plate.

^b^Pair 2: Mother’s knowledge for fruits and vegetables selection on her plate.

^c^Pair 3: Mother’s self-efficacy for fruit and vegetable selection on her plate.

^d^Pair 4: Mother’s stage of change for selecting more fruits and vegetables when preparing family meals.

^e^Pair 5: Mother’s knowledge for selecting more fruits and vegetables when preparing meals.

^f^Pair 6: Mother’s self-efficacy for selecting more fruits and vegetables when preparing family meals.

^g^Pair 7: Mother’s stage of change for selecting more fruits and vegetables outside of the home.

^h^Pair 8: Mother’s level of knowledge for selecting more fruits and vegetables outside of the home.

^i^Pair 9: Mother’s self-efficacy for selecting more fruits and vegetables outside of the home.

^j^Pair 10: Mother’s stage of change for engaging in physical activity.

^k^Pair 11: Mother’s level of knowledge for engaging in physical activity.

^l^Pair 12: Mother’s self-efficacy for engaging in physical activity.

^m^Pair 13: Global stage of change score.

^n^Pair 14: Global knowledge score.

^o^Pair 15: Global self-efficacy score.

**Table 2:** Children’s reasons for recommending cartoon videos.

Emergent themes	Direct quotes
Dietary knowledge gains	“They’re funny and help you learn more about what to eat if you want to eat healthy and stay healthy.”
Dietary knowledge gains	“Because people need to eat better and I think that this is a good way to help people see that it is good to eat fruit and veggies.”
Vicarious learning via character identification	“The people are brown skinned like me.”
Vicarious learning via character identification	“Because they are cartoons and kids can relate to them and it might get the point across about healthy eating.”
Vicarious learning via character identification	“Because some of my friends are fat and they have big bellies.”
Influential for both mother and child	“Because some moms do not want their children to have diabetes.”

**Table 3:** Children’s reasons for not recommending videos.

Emergent themes	Direct quotes
Vicarious learning via an urban environment	“Maybe if the videos talked about why neighborhoods don’t have certain foods. We live in Queens and can go to Fairway, Stop n Shop and Trader Joe’s and even Whole Foods. My friends who live in the school’s neighborhood can’t.”
Vicarious learning via an urban environment	“Just make it more compatible to an urban child’s experience in day to day life situations.”
Vicarious learning via character identification	“Better cartoon people and talk about how we kids may overeat when being bullied.”
Animation quality and graphic design	“Add arms, legs, and necks to the characters, make the voices match the characters, find different ways to show excitement.”

### Mothers’ Feedback and Preferences for Video Improvements

Mothers’ responses were analyzed for emergent themes, and direct quotes are provided—as shown in Table 4. Of the 93 mothers, 68 (73%) found the cartoons to be a good educational resource worth recommending to their peers, thereby diffusing the innovation. Moreover, 37% (34/93) mothers stated the cartoons were educational about eating at home, eating away from home, and cooking with their child. Furthermore, 23% (21/93) mothers said they learned about exercising. A total of 26% (24/93) women commented that the website could have been improved with better graphics and animation. Finally, 16% (15/93) mothers commented that more activities should be included to make the website longer.

**Table 4:** Mothers’ reasons for recommending the website.

Emergent themes	Selected direct quotes
Dialogue via mother and child	“Helps start conversation about nutrition and exercise with children.”
Dialogue via mother and child	“Because watching these videos with my child caused an instant change in my families’ eating habit, but also more importantly in some way created an instant bond that we didn’t know we lacked! We’ll be preparing meals together more frequently and sharing lots of other parent/child activities!”
Dietary knowledge gains	“The website is very informative, I think there is a lot of information to be found on this website on creating healthy choices.”
Diffusion of innovation	“I would recommend it because I believe there are many moms that may not know the various food groups and how to provide proper nutrition.”

## Discussion

### Principal Findings

Our findings indicate that culturally adapted nutrition and physical activity messages can inspire African American mother-child dyads’ readiness to adhere to national dietary and physical activity guidelines. This study aligns with previous research indicating that digital cartoon animation storytelling motivated mothers and children toward dietary and physical activity improvements [15-17,19-21]. Our eHealth intervention extends beyond prior interventions targeting only children and adolescents by incorporating the mother, thereby influencing the larger family unit. Our study was effective in increasing knowledge and self-efficacy in both mother and child. Both mother and child moved across the Stage of Change continuum from not thinking about or considering diet and physical activity improvements (precontemplation or preparation) to considering and carrying out diet and physical activity improvements (preparation or action or maintenance). Given that African American participation in eHealth interventions is low [22], culturally adapted animation may have helped with participation rates.

### Mothers’ Preferences for Culturally Tailored Animation

Our findings reinforce the importance of including mothers in Web-based behavior change interventions because they typically influence the food choice decisions for their families [23,24]. Children’s food preferences into adolescence may be shaped by the foods they are exposed to early in life [25], which may be predisposed by mothers’ own food preferences. Parental role modeling of food intake has also been shown to influence children’s engagement in physical activity and food choices [26-28], which are adjunctive strategies in obesity prevention and management. Dwyer et al found a positive correlation between parent-adolescent dyad autonomous motivation and fruit and vegetable intake [29]. Similarly, Vepsäläinen et al reported that mother-child dietary patterns were stronger than father-child dietary patterns as a significant predictor for the child’s food intake [30]. Targeting the family environment is crucial in inducing adherence to healthy dietary patterns among children and their families [31].

### Comparisons With Prior Work

Prior interventions involving African American parent-child dyads related to healthy eating and physical activity were found in within-person interventions. The FIT Families trial in Michigan conducted from 2011 to 2013 [32] examined satisfaction and content acceptance of a 6-month family-based weight loss intervention. Semi-structured interviews with 136 African American adolescents (median age: 14 years) and caregivers of which 89.7% (122/136) were mothers reported that program components (parent involvement and weight loss counseling) and content (cognitive behavioral skills training, motivational interviewing, and contingency management) were helpful and acceptable. Identified areas of improvement included a need for more engaging skills-based learning, inclusion of exercise sessions, and the need for personalized tailoring to meet adolescent and caregiver interest. Parenting support, active learning, and individualized personalization were positive attributes of the intervention. Personalized tailoring of educational content was consistent feedback among our participants, reinforcing the significance of incorporating features that address interests and issues germane to the target population.

Berry et al evaluated the efficacy of a 5-year cluster-randomized controlled trial in North Carolina on diet and exercise education with coping skills training in elementary school children and their parents [33]. A total of 63.5% (117/184) of participant families were African American, 54.8% (101/184) of whom were mothers. The intervention included an hour of nutrition and exercise coping skills training and 45 min of exercise once a week for 12 weeks. Researchers aimed to examine changes in adiposity, weight, health behaviors, and self-efficacy in children and parents (n=184) in an 18-month program. Intervention group parents reported positive correlations between parent and child waist circumference (*r*=.409; *P*<.001), triceps (*r*=.332; *P*<.001), and subscapular (*r*=.292; *P*<.001) skinfolds compared with controls but failed to find significant correlations in nutrition and exercise self-efficacy and knowledge or change in children’s and parents’ body mass index. Changes in parent-child body weight could be attributed to the consistent physical activity component. Our study effectively increased knowledge and self-confidence to carry out diet and behavior change but does not measure any actual changes in body weight or actual food intake, indicating that an in-person component may help lead to participant weight loss.

Mothers’ open-ended responses regarding video improvements included the preference for the cartoons to better depict food desserts. African American neighborhoods experience the highest rates of unhealthy food environments compared with whites and proportionally the highest rates of obesity and diabetes [34,35]. Character dialogue and backgrounds that illustrate limited access to healthy foods in their neighborhood environment would better reflect the experience of mothers when making food purchasing decisions and proffer possible solutions to overcome this challenge.

### Children’s Preferences for Culturally Tailored Animation

Cartoons are an effective tool for improving children’s adherence to specific health behaviors [36]. Culturally tailored cartoons allowed for positive character identification and vicarious learning for African American children. Open-ended comments from children stating that they liked the videos “because they have brown skin like me,” reflects a positive connection and identification with the characters viewed.

Vicarious learning through brown-skinned characters allows for positive reinforcement of healthful behaviors that is particularly important for African American children to be exposed to and identify with. Viewing brown-skinned cartoon characters is important for instilling self-esteem in children as part of their socioemotional development [37,38]. Evidence suggests children least prefer the color black with a positive happy emotion [39]. Instilling self-confidence in African American youth through vicarious learning could increase self-efficacy toward consuming a more healthful diet.

### Limitations

Stages of Change pre- and posttest surveys assessed readiness toward the target behaviors but not actual consumption. Furthermore, child responses may have reflected the mother’s interpretation as mothers served as a proxy respondent for their children. The study did not include other caregivers, such as grandmothers, who may also make food purchasing decisions and cook meals in the home. The study design assumes target participants have internet access. Moreover, 13% of African Americans do not use the internet [40]. Health literacy [41], internet self-efficacy [42], and parental consent form literacy [43] may have been enrollment barriers. Technological limitations hampered recommending the videos to others. Internet privacy is also a concern, given the degree of mistrust in the black community with the health care system [44].

Low sample size may have affected statistical power during study analysis. Response, social desirability, and selection bias may have been introduced. Methodological improvements could have addressed survey measures that were based on an adapted survey tool. Survey items should be validated for future use. External validity of this study may only apply to the target population.

### Future Directions

Future research should evaluate the role of culturally tailoring as a mediator to influence behavioral outcomes. Explaining how cultural adaptations influence outcomes is critical to understanding their significance [45] and identifying preferable mechanisms for reaching this hard-to-reach population. Animation and content features preferred by this population could be important for future research.

Animation design improvements are needed in the next steps, especially for improving computerized cartoon voices that were a prominent concern in children’s feedback. Privacy concerns and internet access may be areas to address when developing eHealth solutions targeting the African American community [10]. Future study design improvements could include interactive methods to increase engagement, such as photo uploads of before and after pictures of meals consumed and regularly updated website content.

### Conclusions

This study demonstrated that culturally adapted cartoons may be an effective educational tool to inspire healthy behaviors in African American mother-child dyads. Future directions should incorporate key findings from this study for animation design to better reflect the preferences by the target population. Food justice issues commonly experienced in African American communities should be depicted graphically and reflected in character dialogue. Technological advances in mobile apps could draw upon these findings to develop solutions with preferred features by this target population to address their health needs. Self-monitoring tools with goal-setting attributes that have reported successful behavior change may also be considered, including the mother for collective participation and objective measurement.

## Appendix

Multimedia Appendix 1 Screenshot of mother and children meal planning in grocery store.

## Abbreviations

eHealth: electronic health

## References

[1] Centers for Disease Control and Prevention.

[2] Banfield EC, Liu Y, Davis JS, Chang S, Frazier-Wood AC (2016). Poor adherence to US dietary guidelines for children and adolescents in the National Health and Nutrition Examination Survey population. J Acad Nutr Diet.

[3] Gu X, Tucker K (2017). Dietary intakes of the US child and adolescent population and their adherence to the current dietary guidelines: trends from 1999 to 2012. FASEB J.

[4] Ogden CL, Carroll MD, Lawman HG, Fryar CD, Kruszon-Moran D, Kit BK, Flegal KM (2016). Trends in obesity prevalence among children and adolescents in the United States, 1988-1994 through 2013-2014. J Am Med Assoc.

[5] Haughton CF, Wang ML, Lemon SC (2016). Racial/ethnic disparities in meeting 5-2-1-0 recommendations among children and adolescents in the United States. J Pediatr.

[6] Noar S, Harrington N (2016). Tailored communications for health-related decision-making behavior change. Handbook of Health Decision Science.

[7] McElfish P, Post J, Rowland B (2016). A social ecological and community-engaged perspective for addressing health disparities among Marshallese in Arkansas. Int J Nurs Clin Pract.

[8] Cho D, Kim S (2018). Interplay between self-efficacy and perceived availability at home and in the school neighborhood on adolescents' fruit and vegetable intake and energy-dense, low-nutrient food and sugary drink consumption. J Nutr Educ Behav.

[9] Office of Disease Prevention and Health Promotion.

[10] (2018). Pew Research Center.

[11] James DC, Harville C, Whitehead N, Stellefson M, Dodani S, Sears C (2016). Willingness of African American women to participate in e-Health/m-Health research. Telemed J E Health.

[12] Callender C, Thompson D (2017). Text messaging based obesity prevention program for parents of pre-adolescent african american girls. Children (Basel).

[13] Loh IH, Schwendler T, Trude AC, Anderson SE, Cheskin LJ, Lange S, Gittelsohn J (2018). Implementation of text-messaging and social media strategies in a multilevel childhood obesity prevention intervention: process evaluation results. Inquiry.

[14] Burton WM, White AN, Knowlden AP (2017). A systematic review of culturally tailored obesity interventions among African American adults. Am J Health Educ.

[15] Thompson D, Baranowski T, Cullen K, Watson K, Canada A, Bhatt R, Liu Y, Zakeri I (2008). Food, fun and fitness internet program for girls: influencing log-on rate. Health Educ Res.

[16] Baranowski T, Buday R, Thompson DI, Baranowski J (2008). Playing for real: video games and stories for health-related behavior change. Am J Prev Med.

[17] Thompson D, Baranowski T, Cullen K, Watson K, Liu Y, Canada A, Bhatt R, Zakeri I (2008). Food, fun, and fitness internet program for girls: pilot evaluation of an e-Health youth obesity prevention program examining predictors of obesity. Prev Med.

[18] University of Houston.

[19] LeRouge C, Dickhut K, Lisetti C, Sangameswaran S, Malasanos T (2016). Engaging adolescents in a computer-based weight management program: avatars and virtual coaches could help. J Am Med Inform Assoc.

[20] Lu AS, Baranowski T, Hong SL, Buday R, Thompson D, Beltran A, Dadabhoy HR, Chen T (2016). The narrative impact of active video games on physical activity among children: a feasibility study. J Med Internet Res.

[21] Ugalde M, Brand L, Beltran A, Dadabhoy H, Chen T, O'Connor TM, Hughes SO, Baranowski T, Buday R, Nicklas TA, Baranowski J (2017). Mommio's recipe box: assessment of the cooking habits of mothers of preschoolers and their perceptions of recipes for a video game. JMIR Serious Games.

[22] James DC, Harville C, Sears C, Efunbumi O, Bondoc I (2017). Participation of African Americans in e-Health and m-Health studies: a systematic review. Telemed J E Health.

[23] Brand L, Beltran A, Buday R, Hughes S, O'Connor T, Baranowski J, Dadabhoy HR, Diep CS, Baranowski T (2015). Training vegetable parenting practices through a mobile game: iterative qualitative alpha test. JMIR Serious Games.

[24] DeJesus JM, Gelman SA, Viechnicki GB, Appugliese DP, Miller AL, Rosenblum KL, Lumeng JC (2018). An investigation of maternal food intake and maternal food talk as predictors of child food intake. Appetite.

[25] De Cosmi V, Scaglioni S, Agostoni C (2017). Early taste experiences and later food choices. Nutrients.

[26] Ickes S, Mahoney E, Roberts A, Dolan C (2016). Parental involvement in a school-based child physical activity and nutrition program in Southeastern United States: a qualitative analysis of parenting capacities. Health Promot Pract.

[27] Scaglioni S, De Cosmi V, Ciappolino V, Parazzini F, Brambilla P, Agostoni C (2018). Factors influencing children's eating behaviours. Nutrients.

[28] Vaughn AE, Martin CL, Ward DS (2018). What matters most-what parents model or what parents eat?. Appetite.

[29] Dwyer LA, Bolger N, Laurenceau J, Patrick H, Oh AY, Nebeling LC, Hennessy E (2017). Autonomous motivation and fruit/vegetable intake in parent-adolescent dyads. Am J Prev Med.

[30] Vepsäläinen H, Nevalainen J, Fogelholm M, Korkalo L, Roos E, Ray C, Erkkola M, DAGIS consortium group (2018). Like parent, like child? Dietary resemblance in families. Int J Behav Nutr Phys Act.

[31] Suggs LS, Della BS, Rangelov N, Marques-Vidal P (2018). Is it better at home with my family? The effects of people and place on children's eating behavior. Appetite.

[32] Campbell-Voytal K, Hartlieb KB, Cunningham PB, Jacques-Tiura AJ, Ellis DA, Jen KL, Naar S (2017). Evaluation of an evidence-based weight loss trial for urban African American adolescents and caregivers. J Nutr Health.

[33] Berry DC, McMurray RG, Schwartz TA, Hall EG, Neal MN, Adatorwovor R (2018). Correction to: a cluster randomized controlled trial for child and parent weight management: children and parents randomized to the intervention group have correlated changes in adiposity. BMC Obes.

[34] Lee DC, Gallagher MP, Gopalan A, Osorio M, Vinson AJ, Wall SP, Ravenell JE, Sevick MA, Elbel B (2018). Identifying geographic disparities in diabetes prevalence among adults and children using emergency claims data. J Endocr Soc.

[35] Cooksey-Stowers K, Schwartz MB, Brownell KD (2017). Food swamps predict obesity rates better than food deserts in the United States. Int J Environ Res Public Health.

[36] Frémont A, Abou TR, Wanin S, Lebras M, Ollier V, Nathanson S, Hadchouel A, Drummond D (2018). Cartoons to improve young children's cooperation with inhaled corticosteroids: a preliminary study. Pediatr Pulmonol.

[37] McCloskey ML, Thompson DA, Chamberlin B, Clark L, Johnson SL, Bellows LL (2018). Mobile device use among rural, low-income families and the feasibility of an app to encourage preschoolers’ physical activity: qualitative study. J Med Internet Res.

[38] Nelson K (2016). Academic Symposium of Undergraduate Scholarship.

[39] Bubna K, Hegde S, Rao D (2017). Role of colors in pediatric dental practices. J Clin Pediatr Dent.

[40] (2018). Pew Research Center.

[41] Yamashita T, Bardo AR, Liu D, Cummins PA (2018). Literacy, numeracy, and health information seeking among middle-aged and older adults in the United States. J Aging Health.

[42] Karaseva A (2016). Relationship of internet self-efficacy and online search performance of secondary school teachers. Procedia Soc Behav Sci.

[43] Hadden KB, Prince LY, Moore TD, James LP, Holland JR, Trudeau CR (2017). Improving readability of informed consents for research at an academic medical institution. J Clin Transl Sci.

[44] Hamel LM, Penner LA, Albrecht TL, Heath E, Gwede CK, Eggly S (2016). Barriers to clinical trial enrollment in racial and ethnic minority patients with cancer. Cancer Control.

[45] Joseph RP, Keller C, Affuso O, Ainsworth BE (2017). Designing culturally relevant physical activity programs for African-American women: a framework for intervention development. J Racial Ethn Health Disparities.

