# Supplementary figures and images for: Feasibility and Acceptability of a Culturally Tailored Website to Increase Fruit and Vegetable Intake and Physical Activity Levels in African American Mother-Child Dyads: Observational Study
Source: JMIR Pediatr Parent. 2019 Mar 22;2(1):e12501. doi: 10.2196/12501 (PMC6715398; doi:10.2196/12501)

## Appendix

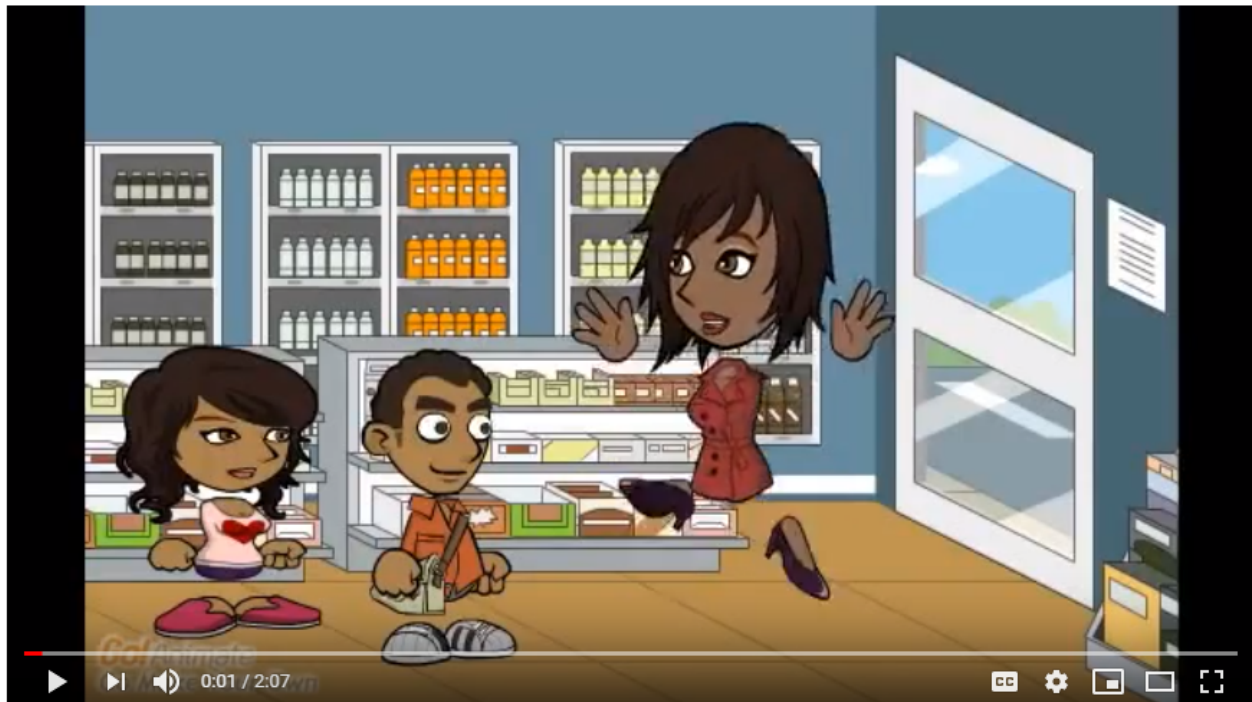

Supplement: Multimedia Appendix 1 [file pediatrics_v2i1e12501_app1.pdf]
